# HIV/AIDS research in Africa and the Middle East: participation and equity in North-South collaborations and relationships

**DOI:** 10.1186/s12992-020-00609-9

**Published:** 2020-09-17

**Authors:** Gregorio González-Alcaide, Marouane Menchi-Elanzi, Edy Nacarapa, José-Manuel Ramos-Rincón

**Affiliations:** 1 Department of History of Science and Documentation, University of Valencia, Valencia, Spain; 2 Department of Internal Medicine, General University Hospital of Alicante, Alicante, Spain; 3 Infectious Disease Division, Carmelo Hospital of Chókwè – Daughters of Charity, Saint Vincent of Paul, Chókwè, Gaza Province Mozambique; 4 Tinpswalo Association, Research Unit, Vincentian Association to Fight AIDS and TB, Chókwè, Gaza Province Mozambique; 5 Department of Clinical Medicine, Miguel Hernandez University of Elche, Alicante, Spain

**Keywords:** Scientific research, Human immunodeficiency virus infection, Acquired immune deficiency syndrome, African countries, Bibliometrics, International collaboration, Leadership

## Abstract

**Background:**

HIV/AIDS has attracted considerable research attention since the 1980s. In the current context of globalization and the predominance of cooperative work, it is crucial to analyze the participation of the countries and regions where the infection is most prevalent. This study assesses the participation of African countries in publications on the topic, as well as the degree of equity or influence existing in North-South relations.

**Methods:**

We identified all articles and reviews of HIV/AIDS indexed in the Web of Science Core Collection. We analyzed the scientific production, collaboration, and contributions from African and Middle Eastern countries to scientific activity in the region. The concept of leadership, measured through the participation as the first author of documents in collaboration was used to determine the equity in research produced through international collaboration.

**Results:**

A total of 68,808 documents published from 2010 to 2017 were analyzed. Researchers from North America and Europe participated in 82.14% of the global scientific production on HIV/AIDS, compared to just 21.61% from Africa and the Middle East. Furthermore, the publications that did come out of these regions was concentrated in a small number of countries, led by South Africa (41% of the documents). Other features associated with HIV/AIDS publications from Africa include the importance of international collaboration from the USA, the UK, and other European countries (75–93% of the documents) and the limited participation as first authors that is evident (30 to 36% of the documents). Finally, the publications to which African countries contributed had a notably different disciplinary orientation, with a predominance of research on public health, epidemiology, and drug therapy.

**Conclusions:**

It is essential to foster more balance in research output, avoid the concentration of resources that reproduces the global North-South model on the African continent, and focus the research agenda on local priorities. To accomplish this, the global North should strengthen the transfer of research skills and seek equity in cooperative ties, favoring the empowerment of African countries. These efforts should be concentrated in countries with low scientific activity and high incidence and prevalence of the disease. It is also essential to foster intraregional collaborations between African countries.

## Background

HIV infection and its clinical manifestation, AIDS, are considered a pre-eminent challenge for global public health [1], affecting populations worldwide since the 1980s. Despite the progress made in prevention and treatment programs, the disease is still pandemic, with the African continent being the hardest hit [2]. An estimated 37.9 million people were living with HIV in 2018, of whom 20.6 million lived in Eastern and Southern Africa, 5 million in Western and Central Africa, and 240,000 in the Middle East and North Africa. The same year saw about 770,000 deaths from this disease and 1.7 million new infections, 61% of which occurred in sub-Saharan Africa. Over half of the new cases in Eastern and Southern Africa were concentrated in Mozambique, South Africa, and Tanzania, while 71% of new infections in Western and Central Africa were in Cameroon, the Côte d’Ivoire, and Nigeria. In the Middle East and North Africa, two-thirds of new cases were registered in Egypt, Iran and Sudan [3]. In response to this challenge, researchers worldwide have worked to produce evidence on HIV/AIDS across a wide range of biomedical disciplines, including epidemiology, virology, immunology, and pharmacology, as well as in non-biomedical fields such as social sciences and the humanities. This body of work has situated HIV/AIDS among the most studied infectious diseases today [4].

Bibliometrics is a method that enables the quantitative and qualitative assessment of scientific research in any area of knowledge, at an individual, institutional, or national level [5]. In that sense, ample literature has been published on bibliometric analyses of HIV/AIDS research since the 1980s [6, 7], including some papers that focus specifically on the regions most affected by the virus and the infection, like Central Africa [8]; sub-Saharan Africa [9]; or on countries like Kenya, Uganda, Nigeria, or Lesotho [10–12]. However, many of these papers were published more than a decade ago and investigated the scientific production in the geographical areas analyzed. In the current context of globalization and predominance of cooperative work, Africans are under-represented in terms of authorship in collaborative research publications. This situation has led some investigators to call for studies that quantify authorship equity [13] and explore North-South relationships in research collaboration [8].

The overarching objective of the present study is to provide an up-to-date description of participation from Africa and the Middle East in the literature on HIV/AIDS published in high-visibility journals, and of the role played by researchers from African countries in publications produced in international collaboration. Our specific research questions were: (1) What was the contribution from Africa and the Middle East, both overall and by country, to the global scientific research output on HIV/AIDS? (2) Is North-South participation balanced international collaboration papers? and (3) Are there differences in the subject-area orientation between publications produced with or without participation from African and Middle Eastern authors on HIV/AIDS research?

## Methods

The methodological process was as follows.

### Identification of global scientific research production on HIV/AIDS

To identify the scientific literature on HIV/AIDs, we used the Medical Subject Headings (MeSH) thesaurus of the National Library of Medicine, selecting all of the descriptors related to HIV, human immunodeficiency related to HIV infection, and the development of vaccines for preventing or clinically treating the immunodeficiency. The final MeSH (plus their variants and synonyms) were: HIV, HIV Infections, Acquired Immunodeficiency Syndrome, and AIDS Vaccines.

Although the MeSH thesaurus is linked to the MEDLINE database, which is freely available through the PubMed platform, we performed a second search of the documents identified in MEDLINE and which were also indexed in the Web of Science Core Collection (WoS-CC) databases. Although this database does not cover all of the documents indexed in MEDLINE/PubMed, it does include all of the institutional affiliations (which MEDLINE started listing only in 2014), making it an ideal source for characterizing scientific production by country and the collaboration from Africa and the Middle East in HIV/AIDS publications during the study period.

The collection of journals in the WoS-CC, moreover, represents the information sources with the highest visibility at an international level. Thus, using that source to calculate our bibliometric study indicators allows a vision of the development of the most relevant and impactful research worldwide.

### Definition of the document sample analyzed

**Fig. 1:**
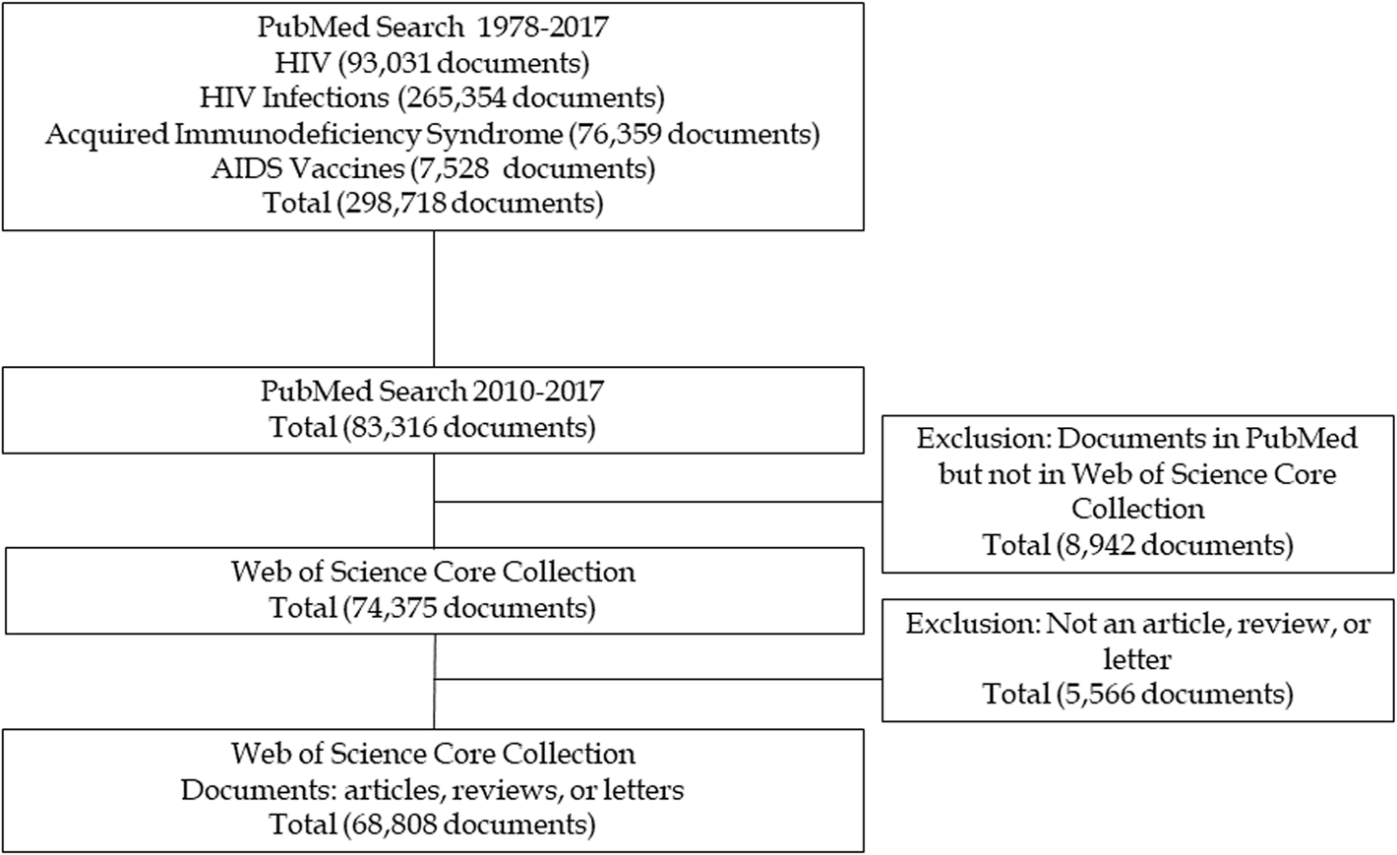
Flow chart for the selection of included documents

Our literature search yielded 93,031 documents on HIV, 256,354 on HIV Infections, 76,359 on Acquired Immunodeficiency Syndrome, and 7528 on AIDS Vaccines. After removing duplicate descriptors, there were 298,718 unique documents. We then restricted the results to those published from 2010 to 2017 (*n* = 83,316) in order to focus the analysis on the most recent research. We ruled out the inclusion of documents from 2018 to avoid delays related to indexation, as at least a year is needed to ensure updated information related to the assignment of MeSH terms. We subsequently identified the documents that were also included in the WoS-CC databases by searching for all of the documents from the initial sample using their PMIDs (the PubMed identifier used as a reference in MEDLINE and included as a bibliographic field in WoS-CC). In total, 89.29% (*n* = 74,375) of the MEDLINE documents were also in the WoS-CC. This set of papers was further restricted to three document types: articles, reviews, and letters (*n* = 68,808), chosen because they are the most prominent papers for transmitting the results of original research (articles); situating and evaluating the development of research in a highly relevant way for other researchers (reviews); and contributing critical viewpoints, comments, relevant information, and perspectives on published studies (letters). The searches took place in November 2018. Figure 1 presents a flow chart showing the selection process for the sample of documents analyzed in the study.

### Download of bibliographic information and review of the standardization of data

Following the bibliographic search and document selection, we downloaded the bibliographic information from the selected records (*n* = 68,808), generating a relational database in Microsoft Access in order to enumerate and individualize the multiple entries contained in certain bibliographic fields. This is the case of institutional affiliations, as a single field collates the data for all co-authors’ institutions and countries. Likewise, the subject area field for the journal of publication may also have several assigned topics, and various MeSH and other text words are assigned to different documents to describe their content.

We also reviewed the standardization and quality of the data. For example, we looked at the years of publication, as the date of some documents’ public dissemination on the journal website differed from the definitive date of publication in the journal (the latter was taken as the reference). Likewise, we consolidated all the information on geographic origins from England, Scotland, Wales, and North Ireland—presented individually in the WoS-CC—under the UK.

### Identification of participation from Africa and the Middle East in HIV/AIDS publications

To analyze the participation from Africa and the Middle East in HIV/AIDS publications, we took as a reference the UNAIDS (2018) definitions of geographical regions, assigning each country to its respective region as defined in that source. The regions were: North America, Western and Central Europe, Asia and Pacific, Eastern and Southern Africa, Latin America and the Caribbean, West and Central Africa, the Middle East and North Africa, and Eastern Europe and Central Asia.

### Indicators obtained and analyses performed

The indicators and analyses applied in our study are structured in three blocks.

#### Analysis of the scientific production, research collaboration and leadership, by geographical region

As an introductory step to understanding global HIV/AIDS research, we quantified absolute scientific production by UNAIDS regions, calculating the number of documents authored by researchers from these areas. Moreover, we assessed inter-regional and international collaboration along with research leadership. The concepts used in the present study are defined as follows:

- International collaboration: joint participation in the authorship of a document by researchers from two or more countries.

- Inter-regional collaboration: joint participation in the authorship of a document by researchers from countries in two or more regions.

- Leadership: the degree of participation as the first author of documents in collaboration (number or % with respect to the total documents produced in collaboration).

Geographical affiliations were based, therefore, on authors’ institutional affiliations. The section on limitations includes an in-depth discussion on the shortcomings of this procedure, which should be considered when interpreting the results.

#### Analysis of research production, collaboration and leadership from countries in Africa and the Middle East

To specifically analyze HIV/AIDS research publications from African and Middle Eastern countries, we determined the number of documents authored by researchers from these countries as well as the proportion of total publications with their participation. With regard to research collaboration and leadership, the absolute and relative values on international collaboration are complemented by a specific analysis of research leadership in the top 10 most productive countries in Africa. Furthermore, a directed collaboration network was generated, representing the main African countries collaborating in global HIV/AIDS research. The nodes represent countries, and the links represent countries’ participation in the first positions of authorship. This visual representation clarifies the position that different countries occupy in the network and the collaborative links that they have established.

#### Subject areas and research fields in global HIV/AIDS research production

We analyzed the research subject areas and fields according to the disciplines that contributed most to scientific production on HIV/AIDS, as identified by means of the subject area classification of scientific journals in the WoS-CC as well as the MeSH descriptors and qualifiers assigned to the documents. To compare research orientations, we present data for global research output, for publications produced solely by researchers from African countries, and publications produced through collaborations between researchers from African countries and others (Africa+global collaboration). Pearson’s correlation coefficient was estimated for these three groupings to determine the affinity between African and global research production.

Finally, a co-occurrence network of MeSH terms was generated to analyze the relationships between them and to identify the specific subject areas or research orientations on HIV/AIDS in Africa and the Middle East.

Pajek and VoSViewer (Version 1.6.8, Center for Science and Technology, Leiden University) software were used to perform all processes (analysis, network generation) and obtain all descriptive indicators.

## Results

### Scientific production by region and degree of international collaboration

Scientific production on HIV/AIDS is dominated by North America (which participated in 55.60% of all documents analyzed) and by Western and Central Europe (35.79%). Together, these regions participated in 82.13% of global scientific research production on HIV/AIDS that was indexed in the sources consulted. For their part, the three regions of Africa and the Middle East participated in 21.61% of the documents, albeit contributions from Eastern and Southern Africa (17.80%) were much higher than those from Western and Central Africa (3.34%) and Middle East and North Africa (1.18%) (Table 1). This limited scientific production contrasts with the high percentages of collaboration observed in these regions; in Eastern and Southern Africa, 82.42% of the papers were published in collaboration with authors from countries in other regions, and in Western and Central Africa, 78.39%. In contrast, 43.22% of the documents from North America were produced in inter-regional collaboration, and 47.99% from Western and Central Europe. Looking only at documents produced with inter-regional collaboration, authors from Africa and the Middle East occupied the first position on just 30 to 36% of the papers, compared to 45% for Western and Central Europe and 54% for North America (Table 1).

**Table 1:** Scientific production on HIV/AIDS, by geographical region (2010–2017)

Geographical area	Total documents		Inter-regional collaborations		First author in inter-regional collaboration	
	N	%	N	% (1)	N	% (2)
North America	38,259	55.60	16,535	43.22	8914	53.91
Western and Central Europe	24,625	35.79	11,817	47.99	5342	45.21
Asia and Pacific	12,473	18.13	6019	48.26	2760	45.85
Eastern and Southern Africa	12,249	17.80	10,096	82.42	3633	35.98
Latin America and the Caribbean	4358	6.33	2073	47.57	724	34.93
West and Central Africa	2300	3.34	1803	78.39	546	30.28
Middle East and North Africa	814	1.18	467	57.37	156	33.40
Eastern Europe and Central Asia	632	0.92	496	78.48	104	20.97
*Total*	*68,808*	*100*	*22,082*	*32.09*	*N/A*	*N/A*

(1) Percentage of documents produced in collaboration by authors from countries in two or more regions, relative to the total number of documents produced with the involvement of at least a country from that region (data in first column); (2) Percentage of documents with a first author from that region, relative to the total number of documents produced in inter-regional collaboration (data in second column)

### Scientific production by country and degree of international collaboration

Research production in Africa and the Middle East is concentrated in South Africa, whose researchers participated in 40.94% of the documents from these regions. At some distance are several other countries from Eastern and Southern Africa: Uganda (12.97%), Kenya (10.71%), Malawi (6.19%) and Tanzania (6.03%). Thirteen other countries show values ranging from 1.32 to 4.73%. Nigeria is the most prominent producer in Western and Central Africa, at 4.59%, while Iran leads production in the Middle East and North Africa (2.02%). Another 45 countries in Africa and the Middle East contributed to less than 1 % of the total research output (Table 2). Among the most productive countries (> 100 documents), Iran, Ethiopia, Nigeria, and South Africa present the lowest degree of international collaboration and the highest participation as first authors. Many of these show values of international collaboration that exceed 90%, with participation as first author under 30%. This situation is similar or even more pronounced in most low-producing countries (Table 2).

**Table 2:** Africa and Middle East scientific production on HIV/AIDS, by country (2010–2017)

Country	UNAIDS region*	Total documents		International collaborations		First author in international collaboration	
		N	% African documents	N	%	N	%
South Africa	E & SA	6063	40.94	4620	76.2	1769	38.29
Uganda	E & SA	1921	12.97	1797	93.55	550	30.61
Kenya	E & SA	1586	10.71	1521	95.9	327	21.5
Malawi	E & SA	916	6.19	865	94.43	214	24.74
Tanzania	E & SA	893	6.03	832	93.17	189	22.72
Zimbabwe	E & SA	700	4.73	672	96	134	19.94
Zambia	E & SA	697	4.71	684	98.13	140	20.47
Nigeria	W & CA	679	4.59	425	62.59	144	33.88
Ethiopia	E & SA	555	3.75	332	59.82	132	39.76
Cameroon	W & CA	421	2.84	363	86.22	111	30.58
Botswana	E & SA	375	2.53	356	94.93	77	21.63
Mozambique	E & SA	303	2.05	293	96.7	80	27.3
Iran	ME & NA	299	2.02	102	34.11	57	55.88
Ghana	W & CA	270	1.82	229	84.81	50	21.83
Rwanda	E & SA	269	1.82	264	98.14	78	29.55
Senegal	W & CA	231	1.56	214	92.64	39	18.22
Côte d’Ivoire	W & CA	225	1.52	206	91.56	40	19.42
Burkina Faso	W & CA	196	1.32	180	91.84	49	27.22
DR Congo	W & CA	119	0.80	106	89.08	27	25.47
Egypt	ME & NA	108	0.73	89	82.41	7	7.87
Saudi Arabia	ME & NA	107	0.72	81	75.7	23	28.4
Namibia	E & SA	100	0.68	95	95	11	11.58
Swaziland	E & SA	98	0.66	95	96.94	10	10.53
Qatar	ME & NA	89	0.60	89	100	38	42.7
Benin	W & CA	79	0.53	75	94.94	6	8
Gambia	W & CA	78	0.53	75	96.15	19	25.33
Gabon	W & CA	76	0.51	68	89.47	17	25
Guinea Bissau	W & CA	69	0.47	69	100	32	46.38
Mali	W & CA	69	0.47	65	94.2	10	15.38
Togo	W & CA	67	0.45	59	88.06	14	23.73
Lesotho	E & SA	58	0.39	57	98.28	17	29.82
Morocco	ME & NA	55	0.37	26	47.27	9	34.62
Lebanon	ME & NA	52	0.35	42	80.77	11	26.19
U Arab Emirates	ME & NA	44	0.30	39	88.64	4	10.26
Guinea	W & CA	36	0.24	31	86.11	5	16.13
Republic of the Congo	W & CA	32	0.22	25	78.13	3	12
Cent Afr Republ	W & CA	23	0.16	20	86.96	4	20
Sudan	ME & NA	23	0.16	21	91.3	5	23.81
Tunisia	ME & NA	23	0.16	10	43.48	5	50
Angola	E & SA	19	0.13	18	94.74	3	16.67
Kuwait	ME & NA	16	0.11	10	62.5	2	20
Oman	ME & NA	16	0.11	10	62.5	2	20
Madagascar	E & SA	15	0.10	13	86.67	1	7.69
Niger	W & CA	15	0.10	15	100	1	6.67
Iraq	ME & NA	13	0.09	10	76.92	0	0
Sierra Leone	W & CA	13	0.09	13	100	2	15.38
Jordan	ME & NA	12	0.08	9	75	4	44.44
Liberia	W & CA	12	0.08	12	100	1	8.33
Libya	ME & NA	12	0.08	7	58.33	3	42.86
Burundi	W & CA	11	0.07	11	100	1	9.09
Chad	W & CA	9	0.06	8	88.89	1	12.5
Cape Verde	W & CA	5	0.03	5	100	2	40
Mauritania	W & CA	5	0.03	5	100	3	60
Mauritius	E & SA	5	0.03	5	100	0	0
Algeria	ME & NA	4	0.03	2	50	1	50
Bahrain	ME & NA	4	0.03	3	75	1	33.33
Equat Guinea	W & CA	4	0.03	4	100	0	0
Syria	ME & NA	4	0.03	4	100	0	0
Yemen	ME & NA	4	0.03	4	100	1	25
Djibouti	ME & NA	2	0.01	2	100	2	100
Somalia	ME & NA	2	0.01	2	100	2	100
Palestinian Ter	ME & NA	1	0.01	1	100	0	0
Sao Tome & Prin	W & CA	1	0.01	0	0	0	0
*TOTAL*	*–*	*14,808*	*100*	*11,964*	*80.79*	*N/A*	*N/A*

E & SA: Eastern and Southern Africa; W & CA: West and Central Africa; ME & NA: Middle East and North Africa. N/A: Not applicable

Generally speaking, African research output on HIV/AIDS is characterized by its cooperative links, particularly with the USA, UK, and other European countries (75 to 93% of the collaborations). However, South Africa also stands out for its intraregional ties, and it has become the main reference for research collaboration on HIV/AIDS, both in Eastern and Southern Africa and among the top 10 most productive African countries. It has collaborated with 34 different countries, led 41.44% of the collaborations, and participated in 35.76% of the papers led by other African countries. Uganda ranks second in terms of collaborative leadership within Africa, albeit with values that are much more modest, having led 14.06% of its collaborative research and participated in 11.11% of papers led by other African countries. The rest of the countries contribute less than 10% to the total collaborative links established. Except for South Africa, Uganda, and a few other countries like Zimbabwe, the collaborative links between different countries in Africa are few and far between, constituting weak and sporadic ties (Table 3).

**Table 3:** Collaboration and leadership of top 10 African countries in research papers on HIV/AIDS (2010–2017)

Collaborative leadership		Collaborations with African countries			Collaborations with non-African countries		
Country	Total collaborations	N countries	N collaborations (%)	Main African collaborators (n collaborations)	N countries	N collaborations (%)	Main non-African collaborators (n collaborations)
South Africa	2810	34	392 (13.95)	Zimbabwe (*n* = 44); Uganda (*n* = 39); Malawi (*n* = 34)	43	2418 (86.05)	USA (*n* = 961); UK (*n* = 566); Switzerland (*n* = 133)
Uganda	896	15	133 (14.84)	South Africa (*n* = 49); Zimbabwe (*n* = 18); Tanzania (n = 16)	27	763 (85.16)	USA (*n* = 309); UK (*n* = 175); Canada (*n* = 40)
Kenya	537	14	94 (17.50)	South Africa (*n* = 41); Uganda (n = 13); Zambia (n = 7)	19	443 (82.50)	USA (*n* = 227); UK (*n* = 77); Canada & Netherlands (*n* = 37)
Malawi	387	14	65 (16.80)	South Africa (*n* = 29); Zimbabwe (n = 9); Uganda (n = 5)	20	322 (83.20)	UK (*n* = 106); USA (*n* = 101); Canada (*n* = 29)
Tanzania	324	13	53 (16.36)	South Africa (n = 15); Uganda (n = 11); Kenya & Zambia (n = 5)	21	271 (83.64)	USA (*n* = 93); UK (n = 49); Sweden (*n* = 35)
Zimbabwe	216	12	54 (25)	South Africa (*n* = 31); Malawi (n = 7); Uganda (n = 5)	18	162 (75)	UK (n = 56); USA (n = 49); Norway (n = 19)
Zambia	257	14	52 (20.23)	South Africa (*n* = 21); Zimbabwe (*n* = 7); Uganda (*n* = 6)	20	205 (79.77)	USA (*n* = 85); UK (*n* = 53); Switzerland (*n* = 14)
Nigeria	190	10	32 (16.84)	South Africa (n = 17); Ghana (n = 4); Kenya. Saudi Arabia & Uganda (n = 2)	20	158 (83.16)	USA (*n* = 89); UK (n = 29); Germany (n = 6)
Ethiopia	194	9	29 (14.95)	South Africa (n = 17); Uganda (n = 3); (Sudan & Kenya n = 2)	21	165 (85.05)	USA (n = 34); Belgium (*n* = 31); UK (*n* = 18)
Cameroon	166	12	42 (25.30)	South Africa (n = 21); Burkina Faso (n = 4); (Côte d’Ivoire & Madagascar *n* = 3)	14	124 (74.70)	France (*n* = 43); USA (*n* = 33); Italy (*n* = 13)
**Non-leadership collaboration**		**Collaborations with African countries**			**Collaborations with non-African countries**		
**Country**	**Total collaborations**	**N countries**	**N Collaborations (%)**	**Main African collaborators (n collaborations)**	**N countries**	**N Collaborations (%)**	**Main non-African collaborators (n collaborations)**
South Africa	2862	25	309 (10.80)	Uganda (n = 49); Kenya (n = 41); Zimbabwe (n = 31)	37	2553 (89.20)	USA (*n* = 1418); UK (*n* = 439); Switzerland (*n* = 134)
Uganda	1248	16	96 (7.69)	South Africa (n = 39); Kenya (n = 13); Tanzania (n = 11)	21	1152 (92.31)	USA (*n* = 741); UK (*n* = 176); Canada (*n* = 69)
Kenya	1194	19	80 (6.70)	South Africa (n = 27); Uganda (n = 16); Botswana & Tanzania (n = 5)	22	1114 (93.30)	USA (*n* = 789); UK (*n* = 95); Canada (n = 83)
Malawi	652	12	65 (9.97)	South Africa (n = 34); Uganda (*n* = 8); Zimbabwe (n = 7)	21	587 (90.03)	USA (*n* = 328); UK (*n* = 122); Italy (n = 34)
Tanzania	643	9	53 (8.24)	South Africa (n = 19); Uganda (n = 16); Botswana (n = 6)	18	590 (91.76)	USA (*n* = 335); UK (n = 95); Denmark (*n* = 26)
Zimbabwe	538	9	88 (16.36)	South Africa (n = 44); Uganda (n = 18); Malawi (n = 9)	20	450 (83.64)	USA (*n* = 216); UK (*n* = 156); Netherlands & Switzerland (n = 17)
Zambia	544	12	66 (12.13)	South Africa (*n* = 28); Kenya (n = 7); Uganda (n = 6)	19	478 (87.87)	USA (*n* = 297); UK (n = 77); Switzerland (*n* = 27)
Nigeria	281	12	50 (17.79)	South Africa (*n* = 27); Uganda (n = 6); Ghana & Kenya (n = 3)	14	231 (82.21)	USA (*n* = 155); UK (n = 27); Netherlands (n = 17)
Ethiopia	200	8	20 (10)	South Africa (n = 7); Botswana & Kenya (n = 3)	15	180 (90)	USA (*n* = 65); Sweden (n = 40); Netherlands (n = 18)
Cameroon	252	10	37 (14.68)	South Africa (n = 22); Burkina Faso & Gabon (n = 3)	15	215 (85.32)	France (*n* = 88); USA (*n* = 73); Germany (n = 13)

**Fig. 2:**
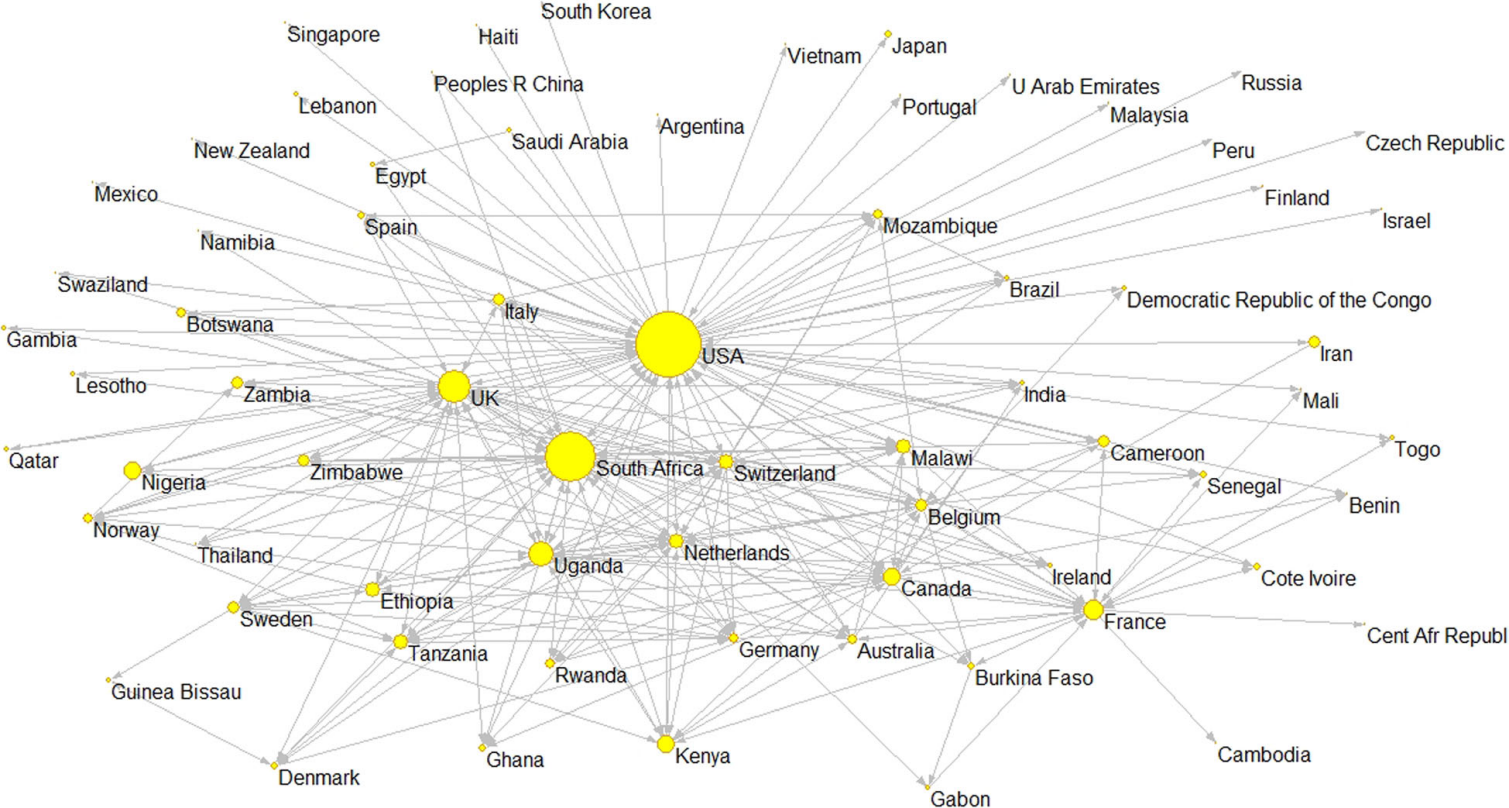
International collaboration network of research papers on HIV/AIDS with African and Middle Eastern countries (2010–2017)

Figure 2 shows a graphic representation of the collaboration network. The USA is in the center as the main reference for international collaboration on scientific output on HIV/AIDS, while the UK, Canada, and other European countries like France, Switzerland, the Netherlands, and Belgium also occupy prominent locations. South Africa is the main African reference for HIV/AIDS publications, reflecting not only its collaborations with the USA, Canada and the European countries but also its prominent role in intraregional collaborations.

### Subject areas addressed in publications on HIV/AIDS in Africa and the Middle East

The correlation analysis on scientific HIV/AIDS output, produced by all countries worldwide, by African countries alone, and through Africa+global collaborations, shows differences in disciplinary orientations and research topics. In terms of disciplines involved, the lowest degree of correlation pertains to global publications versus solely African publications (k = 0.73; Table 4). There is also certain discordance between solely African publications and Africa+global collaborations (k = 0.79). In contrast, there is great affinity between global research output and output from Africa+global collaborations (k = 0.97). Of note, HIV/AIDS publications from Africa alone was dominated by papers in the field of “Public, Environmental & Occupational Health,” while the disciplines of “Infectious Diseases” and “Immunology” occupy the first rankings both globally and in African+global collaborations. The disciplines of “Medicine, General & Internal” and “Health Policy & Services” were also of great relevance in the publications from African countries alone (Table 4).

**Table 4:** HIV/AIDS research papers by Web of Science categories, according to African involvement, 2010–2017

WoS Category	Global publications		Solely African publications		African + global collaborations	
	N	%	N	%	N	%
Infectious Diseases	20,671	21.60	485	17.05	4471	37.37
Immunology	15,369	16.06	311	10.94	3290	27.50
Public. Environmental & Occupational Health	11,853	12.38	742	26.09	2672	22.33
Virology	10,165	10.62	164	5.77	1417	11.84
Multidisciplinary Sciences	5620	5.87	188	6.61	1467	12.26
Microbiology	4989	5.21	60	2.11	824	6.89
Social Sciences. Biomedical	4737	4.95	171	6.01	1023	8.55
Pharmacology & Pharmacy	4155	4.34	62	2.18	380	3.18
Medicine. General & Internal	3473	3.63	278	9.77	623	5.21
Health Policy & Services	2845	2.97	216	7.59	673	5.63
Respiratory System	2657	2.78	168	5.91	752	6.29
Biochemistry & Molecular Biology	2582	2.70	50	1.76	88	0.74
Psychology. Multidisciplinary	1973	2.06	87	3.06	439	3.67
Medicine. Research & Experimental	1872	1.96	41	1.44	204	1.71
Pediatrics	1239	1.29	147	5.17	348	2.91
Health Care Sciences & Services	1207	1.26	98	3.45	311	2.60
Tropical Medicine	1197	1.25	93	3.27	473	3.95
Biotechnology & Applied Microbiology	1075	1.12	40	1.41	111	0.93
Nursing	918	0.96	44	1.55	102	0.85
Obstetrics & Gynecology	775	0.81	94	3.31	197	1.65

Our comparison of the MeSH qualifiers revealed similar disparities (Table 5). The lowest degrees of correlation were between global versus solely African research output (k = 0.68) and between global versus Africa+global collaborations (k = 0.69). However, there was a high degree of correlation between solely African publications and Africa+global collaborations (k = 0.97). With regard to the most prominent MeSH qualifiers, epidemiological studies occupy the top spot in both global and solely African publications. However, “Drug therapy” and “Therapeutic use” are more popular orientations in solely African publications than “Inmmunology,” “Genetics,” and “Metabolism” (Table 5).

**Table 5:** MeSH qualifiers of HIV/AIDS research papers, according to African involvement, 2010–2017

Qualifier	Global publications		Solely African publications		African + global collaborations	
	N	%	N	%	N	%
Epidemiology	53,262	77.41	1220	42.90	5293	44.24
Immunology	49,149	71.43	378	13.29	1805	15.09
Genetics	38,248	55.59	264	9.28	1584	13.24
Metabolism	33,536	48.74	174	6.12	668	5.58
Drug therapy	30,764	44.71	978	34.39	4783	39.98
Therapeutic use	29,749	43.23	681	23.95	3798	31.75
Virology	28,082	40.81	462	16.24	2478	20.71
Complications	25,058	36.42	728	25.60	2338	19.54
Psychology	22,690	32.98	529	18.60	1930	16.13
Prevention & control	18,924	27.50	639	22.47	3212	26.85
Statistics & numerical data	18,496	26.88	498	17.51	2255	18.85
Diagnosis	17,732	25.77	605	21.27	2362	19.74
Drug effects	17,645	25.64	224	7.88	1106	9.24
Blood	15,949	23.18	301	10.58	1262	10.55
Chemistry	15,720	22.85	119	4.18	346	2.89
Administration & dosage	15,703	22.82	218	7.67	1461	12.21
Methods	15,141	22.00	437	15.37	2223	18.58
Pharmacology	14,377	20.89	135	4.75	739	6.18
Pathology	11,124	16.17	186	6.54	654	5.47
Adverse effects	11,122	16.16	213	7.49	798	6.67
Physiology	10,677	15.52	106	3.73	511	4.27
Isolation & purification	8327	12.10	261	9.18	1310	10.95
Transmission	8090	11.76	296	10.41	1546	12.92
Etiology	6038	8.78	249	8.76	579	4.84
Economics	5835	8.48	84	2.95	577	4.82
Therapy	5416	7.87	196	6.89	525	4.39
Microbiology	5377	7.81	181	6.36	718	6.00
Ethnology	5007	7.28	66	2.32	237	1.98
Mortality	4689	6.81	148	5.20	712	5.95
Pharmacokinetics	4033	5.86	21	0.74	220	1.84
Physiopathology	3912	5.69	89	3.13	255	2.13

Finally, with regard to MeSH descriptors, publications from Africa and the Middle East reflects the high prioritization of terms related to prevalence and treatment approaches (Table 6). Furthermore, global scientific production on HIV/AIDS suggests gender parity in terms of the research focus (both the “Male” and “Female” terms were assigned to 55% of the documents). However, for publications produced by researchers from solely African countries, the “Female” term is present in 73.38% of the documents, and for publications produced by Africa+global collaborations, this MeSH appeared in 76.71% of the documents.

**Table 6:** MeSH terms of HIV/AIDS research papers

MeSH Term	Global publications		Solely African publications		African + global collaborations	
	N	%	N	%	N	%
HIV Infections	55,609	80.82	2431	85.48	10,876	90.91
HIV-1	19,945	28.99	378	13.29	2284	19.09
Anti-HIV Agents	12,114	17.61	434	15.26	2647	22.12
Risk Factors	7494	10.89	390	13.71	1525	12.75
Viral Load	6839	9.94	159	5.59	1290	10.78
Antiretroviral Therapy, Highly Active	6758	9.82	327	11.50	1293	10.81
CD4 Lymphocyte Count	6296	9.15	296	10.41	1604	13.41
Prevalence	6172	8.97	463	16.28	1643	13.73
Treatment Outcome	5074	7.37	200	7.03	1091	9.12
Sexual Behavior	4636	6.74	179	6.29	949	7.93
Anti-Retroviral Agents	4529	6.58	196	6.89	1266	10.58
Surveys and Questionnaires	4137	6.01	313	11.01	859	7.18
Acquired Immunodeficiency Syndrome	4047	5.88	253	8.90	524	4.38
Homosexuality, Male	3986	5.79	22	0.77	239	2.00
HIV	3802	5.53	138	4.85	537	4.49
Pregnancy	3728	5.42	287	10.09	1557	13.01
HIV Seropositivity	3604	5.24	241	8.47	787	6.58
RNA, Viral	3497	5.08	48	1.69	467	3.90
Health Knowledge, Attitudes, Practice	3468	5.04	268	9.42	761	6.36
Risk-Taking	3418	4.97	90	3.16	505	4.22
United States	3332	4.84	9	0.32	159	1.33
Coinfection	3238	4.71	204	7.17	673	5.63
South Africa	3197	4.65	832	29.25	2179	18.21
CD4-Positive T-Lymphocytes	3186	4.63	63	2.22	356	2.98
Sexual Partners	3042	4.42	103	3.62	725	6.06
Socioeconomic Factors	2872	4.17	191	6.72	664	5.55
Incidence	2758	4.01	113	3.97	723	6.04
Drug Resistance, Viral	2750	4.00	42	1.48	500	4.18
Virus Replication	2693	3.91	17	0.60	105	0.88
Time Factors	2615	3.80	93	3.27	566	4.73
Genotype	2577	3.75	64	2.25	473	3.95

**Fig. 3:**
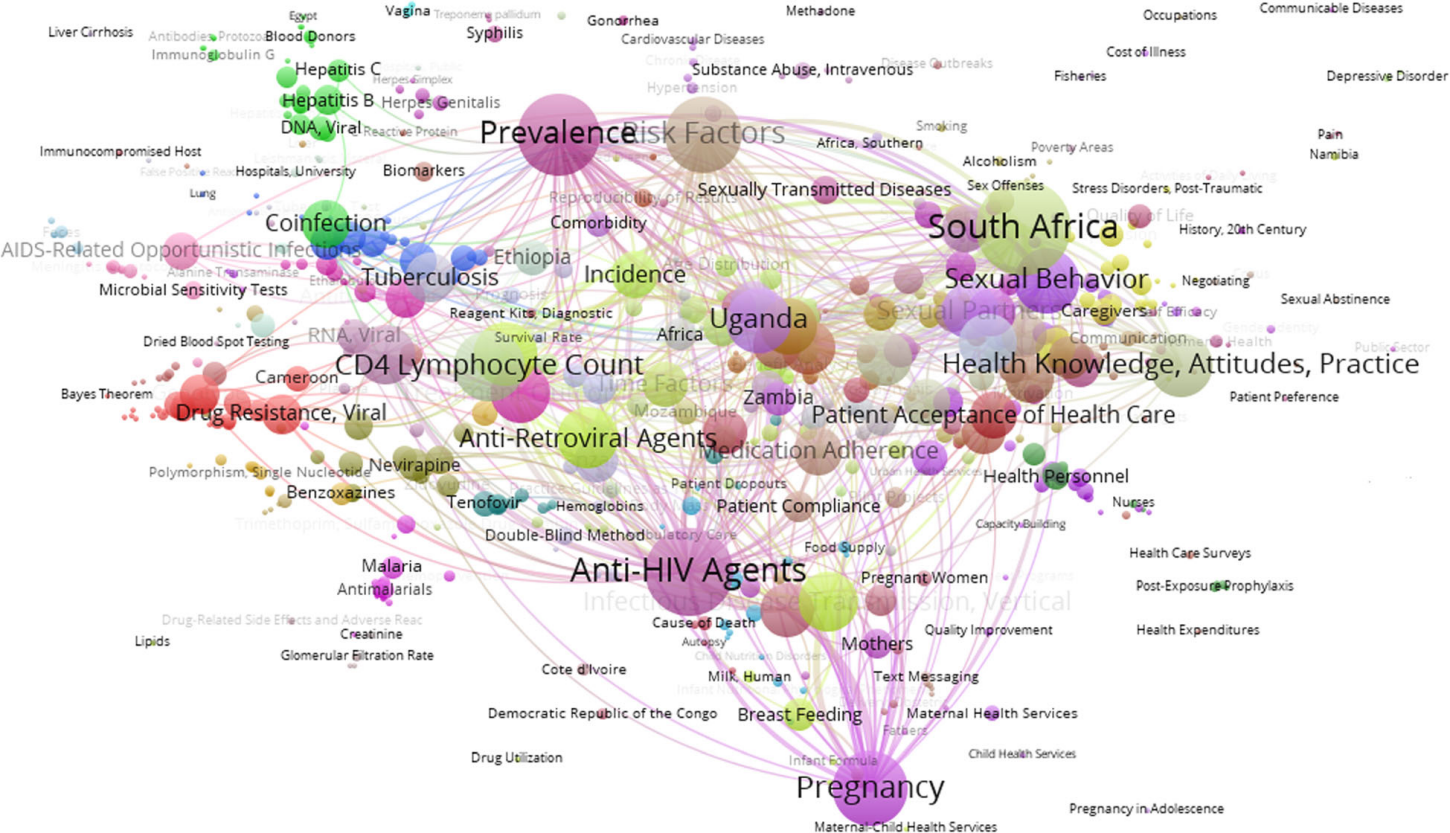
MeSH co-occurrence network on HIV/AIDS research papers from African and Middle Eastern countries (2010–2017)

Figure 3 presents a visualization of the main MeSH terms used to represent Africa and Middle East HIV/AIDS research topics and the links between them. Overall, studies that analyze anti-HIV agents, prevalence, and risk factors constitute the main subject areas that articulate the research. Incidence and its relation to sexual behaviors and health education (knowledge, prevention, acceptance of treatment for the disease) is also an important topic, as is research on pregnancy, maternal health, and prenatal care. Other relevant areas focus on co-infection (with tuberculosis, hepatitis B, hepatitis C, meningitis), resistance to anti-viral agents, and the use of certain medicines to treat the infection (lamivudine, tenofovir etc.).

## Discussion

### Growth, visibility, and concentration of scientific production

Our analysis shows that scientific production on HIV/AIDS is still dominated by researchers from North America and Western and Central Europe, which together participated in 82% of the documents analyzed, although just 6% of people with HIV live in these regions. In contrast, researchers from countries in Africa and the Middle East participated in less than a quarter of the research papers on HIV/AIDS published between 2010 and 2017 (22%), although two-thirds of all people who are infected with the virus live there. Nevertheless, in relation to previous studies analyzing HIV/AIDS publications produced by researchers from African countries, our results indicate two highly relevant trends: (a) the notable growth in scientific production on HIV/AIDS in this region and (b) the elevated participation in scientific publications with greater visibility and international impact. In absolute terms, the number of documents we identified are double those reported by Macías-Chapula & Mijangos-Nolasco [8], based on their analysis of HIV/AIDS literature from sub-Saharan Africa included in the National Library of Medicine from 1980 to 2000, and by Uthman [9] analyzing scientific production on HIV/AIDS from sub-Saharan Africa and indexed in PubMed from 1981 to 2009.

At a country level, the advances made in research are even more significant. In their study on HIV/AIDS literature included in the National Library of Medicine, Onyancha & Ocholla [10] reported negligible contributions from Uganda and Kenya in the form of journal articles published from 1989 to 1998 (*n* = 11 and *n* = 16, respectively). Our results show that these two countries have now become the second and third most productive on the continent, with a high number of contributions to journals indexed in the WoS-CC (*n* = 1921 documents from Uganda and *n* = 1586 in Kenya). Uthman [11] studied HIV/AIDS research production from Nigeria between 1987 and 2006, identifying 254 articles in the WoS databases. Our findings, of 679 documents, nearly triple that number, even though the study period is substantially shorter. In South Africa, the production we identified from 2010 to 2017 (*n* = 6063) is close to that reported by Uthman [9] for the entire period from 1981 to 2009 (*n* = 8361).

Our results also show a trend toward greater research concentration, with an increase in the relative weight of high-producing countries (particularly South Africa, Uganda, and Kenya), which stand out as the main references for African scientific production on HIV/AIDS. Indeed, these countries now account for over half of all publications from Africa, and their relative contributions are trending upward. Thus, while Uthman [9] reported that South Africa participated in 34% of the HIV/AIDS publications produced by sub-Saharan Africa, in our results this figure stands at 43%. Similarly, the relative weight of Uganda and Kenya (the second and third most productive countries) has risen from 8 and 7% of the total contributions, respectively, to 14 and 11%. Similar observations have been made in other research fields [14] and particularly in the biomedical area [15, 16], demonstrating that economic development and investments in research constitute key factors explaining the rise in scientific productivity [17].

The trend toward a greater concentration of research production in a few countries indicates the need to develop policies that facilitate a greater integration of lower-producing and less-developed countries in research activities. The literature describes some measures to stimulate research in these countries that go beyond economic investments, including training and retaining experienced researchers and fostering long-term partnerships based on equitable collaborative research ties. These strategies can enable researchers from these countries to acquire the methodological skills they need and can favor their leadership in spearheading or directing the research [13].

More specifically to the field of HIV/AIDS, Uthman [9] analyzed the factors associated with scientific productivity on HIV/AIDS in sub-Saharan Africa. His results showed that the number of people living with HIV and the number of indexed journals published in the country were predictive of an increase in publications. Other relevant factors include national scientific policies related to countries’ research agendas for this area, plus the adequate integration and participation in the system for publication and dissemination of scientific knowledge. These variables are more closely associated with scientific productivity on HIV/AIDS than others like the number of higher institutions or the number of physicians. The fact that South Africa is the country with the highest number of HIV-positive people and that this subject area has become a priority on the national research agenda [18] is clearly related to the country’s high research productivity in the field. Its economic growth has complemented this boost; together with other BRICS countries, especially China and Brazil, South Africa has laid the groundwork for development by strengthening its educational, healthcare, and social systems [19, 20]. Increased investments in research go hand in hand with this strategy, including through establishing collaborative links with the most advanced economies at a scientific level [21, 22]. However, as Adams et al. [23] signaled in their study, a myriad of factors affect scientific productivity and collaboration in African countries apart from structural factors like the level of economic growth or population size. For example, countries in the Commonwealth sphere, mostly situated in Eastern and Southern Africa and using English as a second language, generally present a higher level of scientific production and collaborative research than other African countries, like those in the Francophone community [16]. Our results are consistent with this trend: 10 of the 12 most productive countries are linked with the Commonwealth.

Although some countries like Nigeria or Ethiopia have made important research efforts, with corresponding increases in their scientific productivity, different studies have highlighted the need for increasing ties with neighboring countries. This would enable a more fluid exchange of knowledge and experience and foster research in key areas like detection and treatment [11, 24].

### High degree of international collaboration, low level of leadership

The two main bibliometric features we observed to be associated with HIV/AIDS research activity in Africa were: (a) a high degree of international collaboration with countries from other geographical regions, dominated by the USA and Europe (81% of the documents) and (b) a low level of research leadership, as seen through the low participation of African investigators as the first authors of documents produced in collaboration (20 to 38% among the top 10 most productive countries).

These two features may reflect a certain scientific dependence and subordination among African countries in relation to more developed countries. Moreover, the same situation has been observed in other biomedical research fields that are of special importance to the global South, like tropical diseases, infectious diseases, and pediatrics [22, 25, 26]. More specifically, Kelaher et al. [27] analyzed randomized controlled trials in the fields of HIV/AIDS, malaria, and tuberculosis that were undertaken in low- and middle-income countries (LMICs) from 1990 to 2013, identifying three relevant features associated with research leadership. First, there was a much higher proportion of first authors from LMICs in studies funded by LMICs (90%) than in studies funded by the USA (32%). Second, participation as first authors from LMICs was sensibly lower in the field of HIV/AIDS (33%) than for other diseases like malaria (67%). Finally, among first authors from all LMICs worldwide, those from Africa authored fewer papers than those from other regions like Latin America or Asia.

The literature describes different barriers that hinder researchers in LMICs from assuming leadership roles. Some of these are related to the absence of infrastructures or adequate financing [28]. Without an established institutional framework, stable research groups cannot be created or sustained; researchers cannot access the technical and financial support they need to submit research tenders; and coordination and monitoring of research priorities in relation to local research agendas is inadequate [13, 29–31]. Other barriers have to do with deficits in methodological skills (like research design and statistical interpretation) or language (composition of articles or fluency in English). All of these factors can affect researchers’ capacity to lead studies and authorship [32–34].

At the same time, there are structural factors related to the hub-and-spoke model that favor the increased recognition and success of countries conducting mainstream research. Economic and human resources are concentrated in North America and Europe, and these regions also establish priority research topics. Editorial bias and the Matthew effect of accumulated advantage cement the structural forces perpetuating the under-representation of researchers from the global South from assuming positions of leadership in scientific publications [26, 32].

The two countries constituting the axis of the collaborative research network on HIV/AIDS are the USA and South Africa. The former stands out for the high number of collaborative links it has established, with its researchers co-authoring papers with most African and Middle Eastern countries (52 countries). In total, 7693 collaborative ties (co-authored papers) were established in the study period, 70% of which were led by researchers in American institutions. Other bibliometric studies have also described the relevance of the USA in collaborative HIV/AIDS research output in Africa [11], Latin America and the Caribbean [35], and Asia [36]. Our own group have highlighted this role in other biomedical research fields [37].

For its part, South Africa is clearly the country of reference for HIV/AIDS research activity on the African continent, with a quantitative weight that is well above that observed in other biomedical areas in which it also exercises leadership. Nachega et al. [16] assessed the participation of African countries in publications on epidemiology and public health in the WoS databases, reporting that South Africa was represented in 22% of the documents, Kenya in 10%, and Nigeria in 9%. In our study, 41% of the documents on HIV/AIDS were authored by researchers in South Africa. This country, along with Ethiopia, is also notable for its leadership, figuring in the affiliations of 38% of the first authors. A similar phenomenon has also been observed in other fields of the health sciences, such as infectious diseases [15, 38].

In addition to maintaining important collaborative ties with the USA and different European countries [39, 40], South Africa has also emerged as a hub for intraregional collaborations within Africa. It has established links with 35 countries—far more than other African countries. Indeed, it is the main collaborator for all the other African countries in the top 10 for HIV/AIDS research productivity, even though these collaborations represent just 12% of the total collaborations in which South Africa participates. In that sense, some papers have called for BRICS countries, including South Africa, to increase their efforts to tackle the challenges primarily affecting the developing world [19]. In the case of South Africa, this could be done by promoting intraregional collaborations in sub-Saharan Africa, as research undertaken at a local level has the most potential to produce benefits, both for population health and socioeconomic development [20, 41]. Hernandez-Villafuerte, Li & Hofman [42] analyzed collaborations among sub-Saharan countries conducting economic evaluations of healthcare interventions, reporting results consistent with ours: researchers in this region tend to collaborate more with Europeans and North Americans than with each other.

The literature highlights specific barriers impeding equitable research collaboration for African researchers, for example the paper by Okeke [43], who pointed to the limited duration of research programs, which should be longer in order to nurture stable collaborations that build hard and leadership capacities. In addition to infrastructure, other aspects mentioned include managerial expertise, administrative capabilities, and the capacity to improvise at African partner institutions. In the same line, Boum II [44] and Boum II et al. [45] discuss the difficulties in harmonizing conflicting interests between Western and African countries, making it essential to prioritize financing for equitable initiatives that lay out specific goals and expectations for partnerships, or which promote initiatives like mentorship programs and investment in Africa-based researchers that strengthen institutional capacity.

Some examples of successful collaborations for promoting equitable research partnerships and African leadership in HIV research include initiatives like the Academic Model Providing Access to Healthcare (AMPATH) in Kenya, the International Epidemiology Databases to Evaluate AIDS (IeDEA) consortium, and different initiatives coordinated and driven by the Africa Centres for Disease Control and Prevention (Africa CDC) or the Joint United Nations Programme on HIV and AIDS (UNAIDS), among others.

### Research interests in public health, epidemiology, and treatment approaches

HIV/AIDS research produced by solely African countries differed from global research in terms of disciplinary and subject area orientations, with a greater focus on public health, epidemiology, and treatment. This finding indicates the need to consider regional, national, and local specificities and interests when determining research priorities. In fact, numerous studies have already signaled the poor alignment between the priorities laid out in African countries’ national research agendas and the research topics that are actually financed [12, 16, 46–49].

From a public health perspective, for example, Uthman [11] pointed out the need for further research evidence to inform HIV prevention and control programs. In this field, some countries perform better than others: South Africa is particularly strong in public health research [50], while other African countries and regions, such as French Africa, have made limited contributions [51].

Studies on epidemiology and treatment approaches for HIV/AIDS are very relevant for research produced in Africa, in contrast to what occurs on a global scale, where these orientations have a relatively limited weight. Nachega et al. [16] pointed out that research on HIV/AIDS, tuberculosis, and malaria have become the main research topics addressed in epidemiological and public health publications in African countries. However, these authors argued for moving epidemiology and public health research beyond the limited sphere of communicable disease control in order to address the regional impact of non-communicable diseases, for example in maternal and child health. This is especially relevant in the case of sub-Saharan Africa, where epidemiologists are overwhelmingly deployed to control infectious diseases, especially HIV/AIDS, tuberculosis, and malaria. The study also calls for strengthening regional expertise in epidemiology in order to shed light on the underlying causes of ill health, rather than to merely control infections and outbreaks [16].

In addition to epidemiological studies, African research also reflects an intense interest in drug therapies for HIV/AIDS, illustrating that control of the infection is a priority for research agendas and policies in African countries [12].

More specifically, previous literature on HIV/AIDS research has shown a greater focus on women in studies carried out with the participation of African researchers [10]. Our study confirms this finding: 73 to 77% of the documents investigated women, compared to 55% in the global literature. One possible explanation for this includes the fact that women are more biologically, economically, socially, and culturally vulnerable to infection. Indeed, for every 10 African men who are HIV-positive, there are 12 to 13 infected women; moreover, 55% of adults who acquire HIV are women, with profound implications for mother-to-child transmission [10]. In consonance with this fact, a greater number of women participate and work on HIV care programs in Africa, and a large proportion of the clinicoepidemiological investigations in these settings are based on care program data [52].

The different epidemiological patterns of HIV/AIDS transmission in North America and Western and Central Europe must also be taken into account, that motivate a greater interest of research in these regions on sexual transmission between men and intravenous drug users. These epidemiological patterns are less important in Africa [53]. The presence in the MeSH co-occurrence network of the descriptors “pregnancy” and “sexual behavior” are noteworthy, reflecting how African researchers are investigating aspects like maternal-fetal transmission of HIV [54] or knowledge and prevention of sexual risk, and changing the preconceptions that still persist about the social determinants of transmission [47]. The prominence of topics related to preventing mother-to-child transmission stands in contrast to the near absence of topics related to children and young people. These groups are especially sensitive to the physical and psychosocial impacts of HIV and AIDS, indicating the need for increased research on young people who are at risk of or living with HIV [55].

The greater research attention to topics related to public health, epidemiology, and treatment may also respond to limited laboratory capacity, which is needed for virologic, immunological, and basic research. In that sense, it is essential to promote initiatives that strengthen these research structures and capacities in African countries, rather than only supporting programs and projects on preventive and clinical approaches.

### Limitations and future lines of research

Limitations of the present study include the fact that a considerable portion of HIV/AIDS research in African countries is disseminated using document types and media that we did not consider, such as meeting abstracts and journals that are not indexed in the WoS-CC. Moreover, using the MeSH thesaurus from the field of health sciences could have resulted in an underestimation of research spheres related to our subject area, such as research in the social sciences. In that sense, some papers have indicated that stigma and discrimination still constitute the main barriers to controlling HIV/AIDS [56]. The process used to assign geographic place variables to the papers included in the sample was based on authors’ stated institutional affiliation; this method has the inherent limitation of not being able to measure the author’s origin, nationality, or identification with the country, but rather the institution’s (and the country’s) capacity to generate outputs in the form of scientific publications. Thus, many researchers of African origin who work at institutions in the USA and Europe would be coded as US/European researchers. Furthermore, the use of first author status as a proxy for African leadership may be misleading, as an African senior author may be the last author on a publication or may have played a leadership role in some aspects other than the manuscript preparation.

Our study focused on obtaining macro indicators on scientific collaboration and output by regions and countries. Future lines of research could conduct meso- or microlevel analyses, for example focusing on the participation of institutions or authors in African HIV/AIDS research or on the impact of the publications. It would also be of great interest to identify the organisms or programs that have funded the research inspiring the publications about HIV, measuring resource contributions according to domestic versus international as well as public versus private origins.

## Conclusions

The main conclusions of our study are as follows.

1. Our results reflect significant progress in African-produced HIV/AIDS research, at both a quantitative level (with notable increases in the number of publications) and qualitative level (through participation in journals indexed in a bibliographic database that brings together the most high-impact and high-visibility international publications). Despite these advances, however, scientific output is still concentrated in a small number of countries, chief among them South Africa, while other countries in Africa and the Middle East make only negligible contributions, despite the high burden of HIV infections.

2. The participation of African countries conducting HIV/AIDS research is characterized by a dependence on and subordination to the USA and European countries. Collaborations between these regions reflect limited leadership by African countries, as measured by the participation of African researchers as the first authors of published studies.

3. HIV/AIDS research conducted with participation from African countries shows appreciably different disciplinary and subject-area interests than global HIV/AIDS research, with a stronger focus on public health, epidemiology, and drug treatments.

It is essential to promote balanced North-South research that properly addresses the most acute needs and gaps in the places where HIV/AIDS has the largest impact. To achieve this balance, it is necessary to transfer research skills to African partners, promote equitable collaborative ties, and empower African countries, especially those with less scientific activity and more disease prevalence. In the same way, the lack of investment in research infrastructure by African governments likely makes it more difficult for African investigators to lead their own research. Intraregional collaborations among African countries can also help to avoid the further concentration of research capacity, reproducing the global North-South model on the African continent.

## Data Availability

The datasets generated and/or analysed during the current study are available in the Harvard Dataverse repository, 10.7910/DVN/RJMAY5.
